# Consolidating the gains and alleviating the pains of a workforce reform: insights from the World Health Organization Africa region's functional review

**DOI:** 10.3389/fpubh.2025.1557143

**Published:** 2025-05-14

**Authors:** Amos Petu, Abdulmumini Usman, Olushayo Oluseun Olu

**Affiliations:** World Health Organization African Regional Office, Brazzaville, Republic of Congo

**Keywords:** workforce reform, WHO African regional office, transformation agenda, WHO functional review, organizational reforms, Africa region

## Introduction

Within the framework of the World Health Organization's (WHO) 13 General Programme of Work (GPW13) and its “triple billion” targets, the African Regional Office (WHO/AFRO) aims to support countries in the African region to ensure that by 2025, an additional 120.6 million Africans will enjoy improved health and wellbeing compared to 2018 (1). Additionally, the WHO/AFRO seeks to ensure that by 2025, ~106 million people gain access to essential services without suffering financial hardship, and 232.3 million are protected from the impact of health emergencies within the same timeframe (1).

Achieving these targets requires an organization that is agile, distinct, and exceptional in the quality of its structure, workforce, operations, and services. Moreover, the importance of fostering a self-evolving organization with a clear competitive stance in resource management and positioning within the health development and humanitarian landscape, considering Africa's significant health challenges, cannot be overstated. Additionally, the increasingly complex development and humanitarian context in the region requires sustained cutting-edge technical assistance which utilizes innovative technologies to improve efficiency, effectiveness, and adaptation to the landscape. The foregoing highlights the importance of a competent and ethical workforce as a critical element in the efficiency and effectiveness of any organization (2).

To achieve its ambitious targets, WHO/AFRO launched a Transformation Agenda (TA) in 2015, with a vision for change and a strategy for institutional regeneration (3, 4). The agenda aimed to deliver products and services that meet the needs and expectations of its Member States (MS) by fostering a culture of excellence, deploying cutting edge technical assistance and aligning organizational resources to member states priorities and ensuring effective partnerships and communication.

As part of its major organizational reform, WHO/AFRO undertook a bold and innovative functional review (FR) and restructuring of its 47 country offices (WCOs) between 2017 and 2020 (5). The review which is the workforce reform stream of the TA sought to ensure an optimal workforce capable of delivering the organization's strategic priorities, improve its managerial practices, and ensure sustainable funding. Not least is a view to improve the functioning of the organization in the areas of governance, policies, procedures, and all its internal systems thereby improving public health outcomes in the Region. The FR represents one of the methods by which WHO/AFRO evolved to embody a distinctive culture and assure that its operational efficiency aligns with the dynamic landscape of health development globally and in the African Region.

In this commentary, we examine the effect of the FR on WHO/AFRO's workforce, managerial practices, and technical cooperation capacity. By analyzing the strengths, weaknesses, opportunities, and threats associated with the FR, we offer recommendations for consolidating and sustaining its outcomes, while also outlining an agenda to address ongoing workforce challenges within the organization. We conclude by highlighting that achieving the objectives set forth in the WHO triple billion objectives will require WHO/AFRO to fully leverage its unique technical and human resources capabilities. This will demand a deliberate focus on utilizing its competitive advantages and harnessing the potential of its workforce, all while incorporating emerging management principles.

## The functional review, its gains and pains

The FR identified four key areas of technical assistance required by WHO/AFRO's MS (4). These are: support to effective coordination of the health sector, generation of evidence to drive prioritization of health activities, health system strengthening, and timely and effective emergency preparedness and response. Nevertheless, the development context of Member States (MSs) varied considerably across the Region, necessitating a tailored approach to ensure the provision of fit-for-purpose technical assistance. To this end, MSs were categorized into four groups based on a set of criteria, including progress toward achieving Universal Health Coverage (UHC), the maturity of national health systems, the presence of public health or humanitarian emergencies, and the status of Disability-Adjusted Life Years (DALYs). Six MSs, characterized by significantly high DALYs, fragile health systems, and the presence of large-scale emergencies, were designated as requiring an operational WHO presence. Conversely, 10 MSs with low DALYs, substantial progress toward UHC, and no major ongoing emergencies were classified as requiring only a strategic WHO presence. The remaining MSs, representing varying levels of need, were categorized as either requiring full technical presence (17 MSs) or moderate technical presence (14 MSs).

Appropriate workforce models to suit each category were also developed and implemented. While such strategic differentiation may not create a mutually exclusive division for countries, it ensured that WHO/AFRO could provide unique and fit-for-purpose technical assistance to its Member States.

Complementary to the FR, a WHO global level organizational reform, which was adopted from the WHO/AFRO TA, also resulted in a more country-focused approach, which aimed to ensure sustainable, predictable, and fit-for-purpose workforce at the country level (6). Within this framework, a task team called the Action Result Group (ARG) was formed to propose actions to further strengthen WHO country offices. The ARG made several proposals to strengthen the country level workforce including a workforce mobility policy and Core Predictive Country Presence (CPCP) model to further ensure that the required workforce is placed at the country level.

The FR achieved several successes (4). First, the introduction of a strategy of mandatory inclusion of one fully qualified female candidate in all recruitment processes resulted in appreciable improvements in the gender balance across all cadre of staff particularly the senior professional cadre with percentage of female staff in the International Professional Officer category (IPO) rising from 25.1% in 2015 to 34.6% in 2024 (7). Second, the introduction of a mentorship initiative as part of expanding the dynamic capability of the organization, led to an increase in younger cadre of staffing, for example junior professional officers and United Nations volunteers (8). Third, the introduction of new staffing cadre such as deputy WHO Representatives, Programme Management Officers, Multi Country Assignment Teams (MCATs) and External Relation Officers in selected countries offices culminated in improvements in the capacity and management of the WCOs, increased partnerships, resources mobilization and visibility of the organization (9). Fourth, the introduction of leadership training and mentoring initiatives equipped senior leaders and mid-level staff members with the skills required to manage their work and programmes more effectively (5).

Nevertheless, the FR came with challenges which are inherent to major workforce reforms. It stretched over a long period with the associated heavy demand on the organization's financial and human resources. Generally, it is desirable that transformation, as a change that leads to improvement in the organization, concludes in good time for impact and minimizes unintended consequences such as widespread job insecurity, demotivation, and poor commitment.

The longer the process, the more apparent the differences in interests and goals of individuals that make up the organization can become, and these conflicts can negatively influence organizational behavior.

## The unfinished workforce reform agenda in World Health Organization Africa region

While several benefits have accrued from the implementation of the FR, several workforce challenges persist in the organization. First, while the FR has attracted a younger cadre of workforce, the clarity about their seamless career transition to managerial positions is unclear. Experiences and models from other organizations that have created such pathways could be useful in addressing this challenge (10). Second, the advent and application of digital technologies to public health also requires a technologically savvy workforce. It is anticipated that internal capacity development will build the requisite staff technological competencies. Hence the need for deliberate opportunities to upgrade staff capacity in this regard. Third, by design, there are limited opportunities for career growth in the organization as staffs are appointed to contract positions with defined characteristics and duration. These could be unfavorable contractual arrangements to staff who would prefer long-term, secure and tenured jobs, thus resulting in inadequate staff commitment and high turnover rate. Fourth, lack of systematic staff succession planning and high turnover rate may result in a lack of institutional memory in programmes. Fifth, it is crucial to periodically define the boundaries of responsibility yet ensure synergy between the MCATs and country level CPCP deployments that both act on the overall behalf WHO/AFRO at the country level. Sixth, the organizational staff performance and evaluation system is inadequate in objectively addressing staff performance. Lastly, the aspects of the FR which should nurture a process of institutionalization of a change in culture will need to be fostered.

## Moving forward: consolidating the gains of the functional review and addressing the outstanding workforce challenges

It is critical for organizations to deploy dynamic capabilities to adapt and succeed in challenging and changing environments. These capabilities involve integrating, building, and reconfiguring internal and external competencies to respond to rapidly evolving circumstances. In the context of WHO/AFRO, aligning its country offices requires sustained leveraging of these dynamic capabilities to enhance operational performance and drive organizational reforms (11). Hence, the gains from the FR should continue to be nurtured and used as a foundation to strengthen the organization's workforce capacity.

Specifically, WHO/AFRO should continue reviewing, refining, and improving the WCO categorization model to ensure that MSs receive fit-for-purpose and context-specific technical assistance. The newly introduced staff cadres should be fully integrated into the system, while additional investment is needed to establish a systematic approach for recruiting, training, mentoring, and gradually promoting young professionals into senior roles. Improving career pathways and opportunities to appropriately reward high-performing staff, while mentoring and guiding low performers, will be critical. Strengthening the elevation of skills and knowledge as central to capacity building should be synchronized with the establishment of a more objective staff performance evaluation system that fosters high performance.

Additionally, staff recruitment procedures should include identifying and head-hunting top performers in the employment market, along with establishing a staff succession plan. This will enhance the staff's ability to interpret and understand the external environment, including institutional rules, norms, and expectations. Such measures will also promote a system that ingrains the organization's values on all staff, reinforcing their belief in its goals, objectives, and principles (12).

## Conclusion

We believe the FR has produced several positive results within WHO/AFR's country offices. Therefore, its benefits should continue to be nurtured to full realization and sustained. The FR should not be seen merely as a process of incidental change but rather as a long-term developmental framework that empowers the organization and its workforce to achieve its strategic goals effectively. However, there remain several unfinished structural challenges in staff career pathways, contractual arrangements and performance management that need to be addressed to ensure optimal organizational performance. In this regard, we advocate for the development of strategies aimed at consolidating and sustaining the gains of the FR, while also addressing the outstanding issues outlined earlier. This can be accomplished within existing frameworks, such as the WHO Regional Committee for Africa's resolution on strengthening country presence for universal health coverage. Additionally, we believe that the recommendations provided in this commentary will contribute meaningfully to the ongoing discourse on this issue. We urge the WHO/AFR leadership to offer the necessary guidance and create an enabling environment to support these efforts.
